# Neighborhood disadvantage and cardiovascular health in early adolescents in the United States

**DOI:** 10.1016/j.ajpc.2026.101749

**Published:** 2026-07-17

**Authors:** Christiane K. Helmer, Olivia Veira, Erin E. Dooley, Hoda S. Abdel Magid, Alexander Testa, Kelley Pettee Gabriel, Fiona C. Baker, Holly C. Gooding, Jason M. Nagata

**Affiliations:** a Division of Adolescent & Young Adult Medicine, Department of Pediatrics, University of California, San Francisco, CA, USA; b Department of Medicine, Brigham and Women's Hospital, Boston, MA, USA; c Department of Epidemiology, University of Alabama at Birmingham, Birmingham, AL, USA; d Department of Population and Public Health Sciences, University of Southern California, Los Angeles, CA, USA; e Department of Management, Policy and Community Health, University of Texas Health Science Center at Houston, Houston, TX, USA; f Center for Health Sciences, SRI International, Menlo Park, CA, USA; g Department of Pediatrics, Emory University, Atlanta, GA, USA

**Keywords:** Teen, Youth, Social determinants of health, Built environment, Cardiometabolic

## Abstract

**Introduction:**

Adolescence is a critical period during which exposures to cardiovascular disease risk factors and related pathophysiologic changes begin. Yet neighborhood-level factors shaping cardiovascular health from adolescence onward remain understudied. The American Heart Association’s Life’s Essential 8 (LE8) is a composite cardiovascular health score, encompassing diet, physical activity, body mass index, blood pressure, and other factors. Area Deprivation Index (ADI), Social Vulnerability Index (SVI), and Childhood Opportunity Index (COI) estimate neighborhood disadvantage. This study examines associations between neighborhood disadvantage and LE8 cardiovascular risk factors in early adolescents.

**Methods:**

We analyzed data from the Adolescent Brain Cognitive Development (ABCD) Study, including 8,160 early adolescents with behavioral subcomponent scores and 1,125 with overall LE8 and health factors subcomponent scores. Neighborhood disadvantage indices (ADI, SVI, COI) were derived from geospatial data. Multivariable linear regression models assessed associations between ADI, SVI, and COI at baseline (2016–2018) and LE8 scores at Year 2 (2018–2020) and Year 3 (2019–2021), adjusted for age, sex, and race/ethnicity.

**Results:**

Higher neighborhood disadvantage was associated with lower (less healthy) LE8 cardiovascular health scores in early adolescents, driven mainly by behavioral factors. Overall LE8 scores were 9.45 points lower for the most deprived ADI quintile (95% CI: -11.86, -7.05; p<0.001), 6.95 points lower for the most vulnerable SVI quintile (95% CI: -9.09, -4.82; p<0.001), and 8.45 points lower for the lowest opportunity COI quintile (95% CI: -10.77, -6.12; p<0.001).

**Conclusion:**

Higher neighborhood deprivation was associated with poorer LE8 cardiovascular health scores in early adolescence.

## 1 Introduction

Cardiovascular disease (CVD) has been the leading cause of death in the United States for decades [1,2]. Although there have been investments in medical therapies for prevention and treatment, CVD remains responsible for one in five deaths annually. Though CVD mortality overwhelmingly affects adults, the underlying disease processes behind atherosclerosis, including endothelial dysfunction, dyslipidemia, and inflammatory cascades, originate in childhood [3,4]. Evaluating cardiovascular risk and addressing its upstream determinants in early adolescence may therefore be a powerful strategy for preventing or delaying the onset of overt disease in adulthood.

The American Heart Association’s (AHA) Life’s Essential 8 (LE8) score provides an evidence-based framework for assessing cardiovascular health by integrating diet, physical activity, sleep, nicotine use, body mass index (BMI), blood lipids, blood glucose, and blood pressure [5]. LE8 has been extensively applied in adult populations, with evidence demonstrating a significant association between higher (i.e., healthier) LE8 scores and reduced CVD risk and mortality [6]. However, fewer studies have applied the framework in adolescents.

Neighborhood context has been increasingly recognized in the literature as a key determinant of cardiovascular risk. Significant evidence demonstrates that neighborhood disadvantage is associated with poorer cardiovascular outcomes in adults [[7], [8], [9]]. In early adolescence, neighborhood characteristics, such as access to healthy food, safe recreational spaces, and limited exposure to environmental hazards, influence the behavioral factors that shape cardiovascular disease risk [10]. Early adolescent CVD risk is predictive of adult cardiovascular disease risk [11,12], making it a useful tool for identifying those who could benefit from early intervention. Recent studies have examined the relationship between neighborhood disadvantage and cardiovascular disease risk among children and adolescents with mixed results [[13], [14], [15]]. Building on this work, the present study uses multiple validated indices of neighborhood disadvantage (e.g., the Area Deprivation Index [16], Social Vulnerability Index [17], and Childhood Opportunity Index [18]) to examine associations between neighborhood resource access and LE8 cardiovascular health scores among early adolescents in the Adolescent Brain Cognitive Development (ABCD) Study [19]. We hypothesize that increased neighborhood disadvantage will be associated with lower LE8 scores (poorer cardiovascular health).

## 2 Methods

We analyzed baseline (2016-2020), Year 2 (2018-2020), and Year 3 (2019-2021) data from the ongoing ABCD Study. The follow-up years were selected for analysis based on data availability of the LE8 components. The ABCD Study, which includes 21 sites across the United States, is the largest longitudinal study of brain health and development in the country [19]. At baseline, 11,875 children (ages 9-10 years) were recruited.

The methodology of the ABCD Study is described in detail elsewhere [20]. Centralized institutional review board (IRB) approval was acquired by the University of California, San Diego and secondary data analysis approval was given by the University of California, San Francisco. A parent or guardian and the child gave written informed consent and assent to the ABCD research team.

### 2.1 Measures

Using the geospatial location data outlined by the Linked External Data Environmental workgroup, the ABCD Study gathers individual, neighborhood, and state-level information needed to calculate neighborhood disadvantage indices [21]. We analyzed three indices of neighborhood disadvantage at baseline: the Area Deprivation Index (ADI), the Social Vulnerability Index (SVI), and the Childhood Opportunity Index (COI). Although these indices are conceptually related, each was developed for a distinct purpose and captures different dimensions of neighborhood context. Specifically, ADI reflects neighborhood socioeconomic deprivation, SVI assesses social vulnerability to external stressors, and COI measures resources and opportunities that support healthy child development. Therefore, all three indices were examined as complementary measures of neighborhood disadvantage and opportunity, with further details on each index provided below.

Area Deprivation Index. The ADI, developed by Gopal Singh, is one of the most robustly validated and utilized tools for measuring neighborhood-level disadvantage in the United States [16]. The index includes 17 indicators from census data, such as the percentage of families below the poverty level, income disparity, median home value, and the number of people aged 25 and older with at least a high school diploma [21]. Our study used baseline ADI percentiles at the census-tract level that included all 17 indicators. In accordance with national ADI percentiles, the 1^st^ quintile represents the lowest neighborhood disadvantage, and the 5^th^ quintile indicates the greatest neighborhood disadvantage.

Social Vulnerability Index. The Centers for Disease Control and Prevention (CDC) developed SVI as a place-based index to assess which communities may need high levels of support before and after stressors such as natural disasters and disease outbreaks [17]. The SVI is derived from county and census tract data and reflects 15 variables from the American Community Survey, organized into four themes, including socioeconomic status, household composition, minority status and language, and housing type and transportation. The resulting index characterizes a community’s vulnerability to severe weather events and public health emergencies [17]. Our study used census-tract-level data at baseline with participants categorized into quintiles based on national SVI percentiles. The 1^st^ quintile represents the lowest vulnerability, and the 5^th^ quintile represents the highest.

Childhood Opportunity Index. The COI 3.0 is a composite index developed to measure children’s access to resources in every neighborhood (at the census-block-level) in the United States [22]. Scores for overall COI and its three domains (education, health and environment, and social and economic opportunities) are included in the ABCD Study. At baseline, participants were categorized into quintiles reflective of national COI percentiles [23]. The 1^st^ quintile represents the lowest neighborhood opportunity, and the 5^th^ quintile represents the highest.

Cardiovascular health was measured using the LE8 score, a composite metric developed by the AHA to promote and assess heart health. LE8 includes diet, physical activity, nicotine exposure, sleep duration, BMI, blood lipids, blood glucose, and blood pressure. All LE8 components were assessed at the Year 2 follow-up, which served as the primary outcome timepoint for this study. To maximize completeness of cardiometabolic outcome data that were missing because of COVID-19-related restrictions on nonessential research activities, corresponding Year 3 measurements were used when Year 2 measurements were not available. Specifically, blood samples were first collected in Year 2 from a subset of participants who consented. However, nearly half of the sample did not complete a blood draw due to COVID-19 restrictions; in Year 3, some participants who missed the Year 2 blood draw were offered this assessment. As a result, blood glucose and lipid measures were available for fewer than 2,000 participants, of whom approximately 25% had Year 3 data imputed because Year 2 data were unavailable. The same approach was used for anthropometric measures (height and weight for BMI) and blood pressure, which were also affected by COVID-19-related restrictions during Year 2.

We computed an LE8 score (measured from 0 to 100), adapted for the ABCD Study based on the LE8 scoring framework previously applied to the National Health and Nutrition Examination Survey (NHANES) [24]. Each of the eight components was assigned a score, and the overall score was calculated as the average of these components. Subcomponent scores were also calculated: the behavioral factors subcomponent score was the average of diet, physical activity, nicotine exposure, and sleep scores. The health factors subcomponent score was the average of the BMI, blood lipids, blood glucose, and blood pressure scores. The health factors score was primarily based on Year 2 data, supplemented with Year 3 measurements for participants who missed the Year 2 follow-up. Higher LE8 scores indicate more favorable cardiovascular health and lower LE8 scores indicate poorer cardiovascular health.

Details of LE8 scoring are described in our previous study [25], which operationalized LE8 in the ABCD Study. For the present study, continuous LE8 scores were used as the primary outcome. In sensitivity analyses, LE8 scores were dichotomized into low–moderate (0–79) and high (80–100), as the small number of participants in the low category precluded use of a three-category classification as typically used in adult studies [26].

### 2.2 Statistical analysis

Of the 11,875 children recruited at baseline, 3,803 had missing behavioral data, missing neighborhood disadvantage data, or missing sociodemographic data. As a result, our study sample included 8,160 early adolescents with behavioral subcomponent scores, and 1,125 children had overall LE8 and health subcomponent scores. Sociodemographic differences between included and excluded participants for each analytic sample are presented in Supplementary Tables S1 and S2.

We calculated descriptive statistics, including means, standard deviations, and percentages, for all independent and dependent variables. Pearson correlation coefficients were calculated to assess overlap among the neighborhood quality indices. We used baseline neighborhood disadvantage (ADI, SVI, and COI) indices as primary predictors, and LE8 cardiovascular health scores at Years 2 and 3 were used as outcomes. We used multivariable linear regression models to estimate associations between neighborhood disadvantage and LE8 scores, adjusting for baseline sociodemographic confounders: age, sex, and race/ethnicity. Statistical significance was assessed using the Benjamini-Hochberg procedure to adjust for false discovery rate given multiple statistical tests. A test for trend was also conducted to assess dose-response relationships among outcomes that were significantly associated in linear regression analyses. For sensitivity analyses, we used modified Poission regression models to estimate associations between neighborhood disadvantage and binary LE8 scores, using high LE8 (80–100) as the reference group. Analyses were conducted using Stata 18 (StataCorp, College Station, TX).

## 3 Results

Sociodemographic characteristics of the 8,160 included participants are represented in Table 1. Of the analytical sample, the average age was 9.9, 47.3% were female, 41.6% were non-White, and the average overall LE8 score was 78.3. Compared to those included in the analysis of overall LE8 scores, those excluded were more likely to be younger and Black (Supplementary Table S2).

**Table 1:** Sociodemographic characteristics at baseline (2016–2018) and Life's Essential 8 scores at Years 2 and 3 (2018–2021) of 8,160 Adolescent Brain Cognitive Development (ABCD) Study analytical sample participants.

Characteristics	Mean (SD) / (%)
Age (years)	9.9 (0.6)
Sex	
Female	47.3%
Male	52.7%
Race and ethnicity	
Asian	6.2%
Black	15.9%
Latino/Hispanic	15.0%
Native American	3.5%
Other	0.9%
White	58.4%
Life's Essential 8 score	
Overall summary score	78.3 (10.4)
Low (0–49)	0.4%
Moderate (50–79)	51.7%
High (80–100)	47.9%
Behavioral factors subcomponent score	67.1 (15.9)
Low (0–49)	0.4%
Moderate (50–79)	19.8%
High (80–100)	79.8%
Health factors subcomponent score	88.5 (11.6)
Low (0–49)	12.7%
Moderate (50–79)	61.1%
High (80–100)	26.2%

SD = standard deviation. The four behavioral factors include diet, physical activity, nicotine exposure, and sleep; the four health factors include body mass index, blood lipids, blood glucose, and blood pressure.

Pearson correlation analyses demonstrated moderate to strong correlations between neighborhood indices (ADI vs. SVI: r = 0.57; COI vs. ADI: r = -0.69; COI vs. SVI: r = -0.84), indicating substantial but incomplete overlap.

Table 2 presents the multivariable linear regression models examining associations between children’s neighborhood quality at baseline and LE8 cardiovascular characteristics at Years 2 and 3. Greater neighborhood disadvantage, as indicated by higher ADI, higher SVI, and lower COI, was associated with lower LE8 cardiovascular health scores among early adolescents (N=1,125 for overall LE8, Fig. 1). Compared with the most advantaged quintile (Q1 for ADI/SVI; Q5 for COI), adolescents in the most disadvantaged quintile had significantly lower overall LE8 scores: ADI (-9.45 points; 95% CI: -11.86, -7.05; p<0.001), SVI (-6.95 points; 95% CI: -9.09, -4.82; p<0.001), and COI (-8.45 points; 95% CI: -10.77, -6.12; p<0.001). Similar findings were observed for the behavioral factors subcomponent score (N=8,160). Adolescents in the most disadvantaged quintile had significantly lower scores compared with those in the most advantaged quintile across all indices: ADI (-13.00 points; 95% CI: -14.27, -11.72; p<0.001), SVI (-8.87 points; 95% CI: -10.04, -7.69; p<0.001), and COI (-11.66 points; 95% CI: -12.85, -10.47; p<0.001). In contrast, associations between neighborhood disadvantage and the health factors subcomponent score were largely nonsignificant (N=1,125).

**Table 2:** Associations of baseline indices of children's neighborhood quality and Life's Essential 8 (LE8) cardiovascular health characteristics at Year 2 and 3 in the Adolescent Brain Cognitive Development (ABCD) Study.

Children's neighborhood quality	Overall LE8 summary score		Behavioral factors subcomponent score		Health factors subcomponent score	
	N=1,125		N=8,160		N=1,125	
	Adjusted B (95% CI)	p	Adjusted B (95% CI)	p	Adjusted B (95% CI)	p
Area Deprivation Index quintile						
1st (lowest)	reference		reference		reference	
2nd	**-2.78 (-4.28, -1.27)**	**<0.001**	**-2.51 (-3.35, -1.68)**	**<0.001**	-1.24 (-3.03, 0.54)	0.173
3rd	**-6.36 (-8.07, -4.65)**	**<0.001**	**-7.14 (-8.14, -6.15)**	**<0.001**	**-2.70 (-4.73, -0.67)**	**0.009**
4th	**-7.37 (-9.56, -5.19)**	**<0.001**	**-11.16 (-12.37, -9.94)**	**<0.001**	-1.59 (-4.18, 1.00)	0.229
5th (highest)	**-9.45 (-11.86, -7.05)**	**<0.001**	**-13.00 (-14.27, -11.72)**	**<0.001**	-2.50 (-5.35, 0.35)	0.086
Social Vulnerability Index quintile						
1st (lowest)	reference		reference		reference	
2nd	**-2.12 (-3.60, -0.64)**	**0.005**	**-1.17 (-2.06, -0.28)**	**0.010**	-1.58 (-3.31, 0.15)	0.073
3rd	**-5.37 (-7.08, -3.65)**	**<0.001**	**-4.72 (-5.72, -3.73)**	**<0.001**	**-3.81 (-5.82, -1.81)**	**<0.001**
4th	**-6.15 (-8.14, -4.16)**	**<0.001**	**-7.23 (-8.33, -6.13)**	**<0.001**	-2.12 (-4.43, 0.20)	0.074
5th (highest)	**-6.95 (-9.09, -4.82)**	**<0.001**	**-8.87 (-10.04, -7.69)**	**<0.001**	**-4.23 (-6.71, -1.74)**	**0.001**
Child Opportunity Index quintile						
5th (highest)	reference		reference		reference	
4th	**-2.93 (-4.37, -1.49)**	**<0.001**	**-3.69 (-4.53, -2.85)**	**<0.001**	-1.38 (-3.07, 0.31)	0.109
3rd	**-3.16 (-4.92, -1.40)**	**<0.001**	**-5.58 (-6.61, -4.55)**	**<0.001**	-1.28 (-3.34, 0.79)	0.224
2nd	**-6.92 (-8.91, -4.93)**	**<0.001**	**-9.20 (-10.37, -8.02)**	**<0.001**	**-2.66 (-5.00, -0.33)**	**0.025**
1st (lowest)	**-8.45 (-10.77, -6.12)**	**<0.001**	**-11.66 (-12.85, -10.47)**	**<0.001**	**-3.54 (-6.26, -0.81)**	**0.011**

**Bold** indicates statistical significance after the Benjamini-Hochberg procedure. B = coefficient. Linear regression models were adjusted for age, sex, and race and ethnicity. The four behavioral factors include diet, physical activity, nicotine exposure, and sleep; the four health factors include body mass index, blood lipids, blood glucose, and blood pressure.

**Fig. 1:**
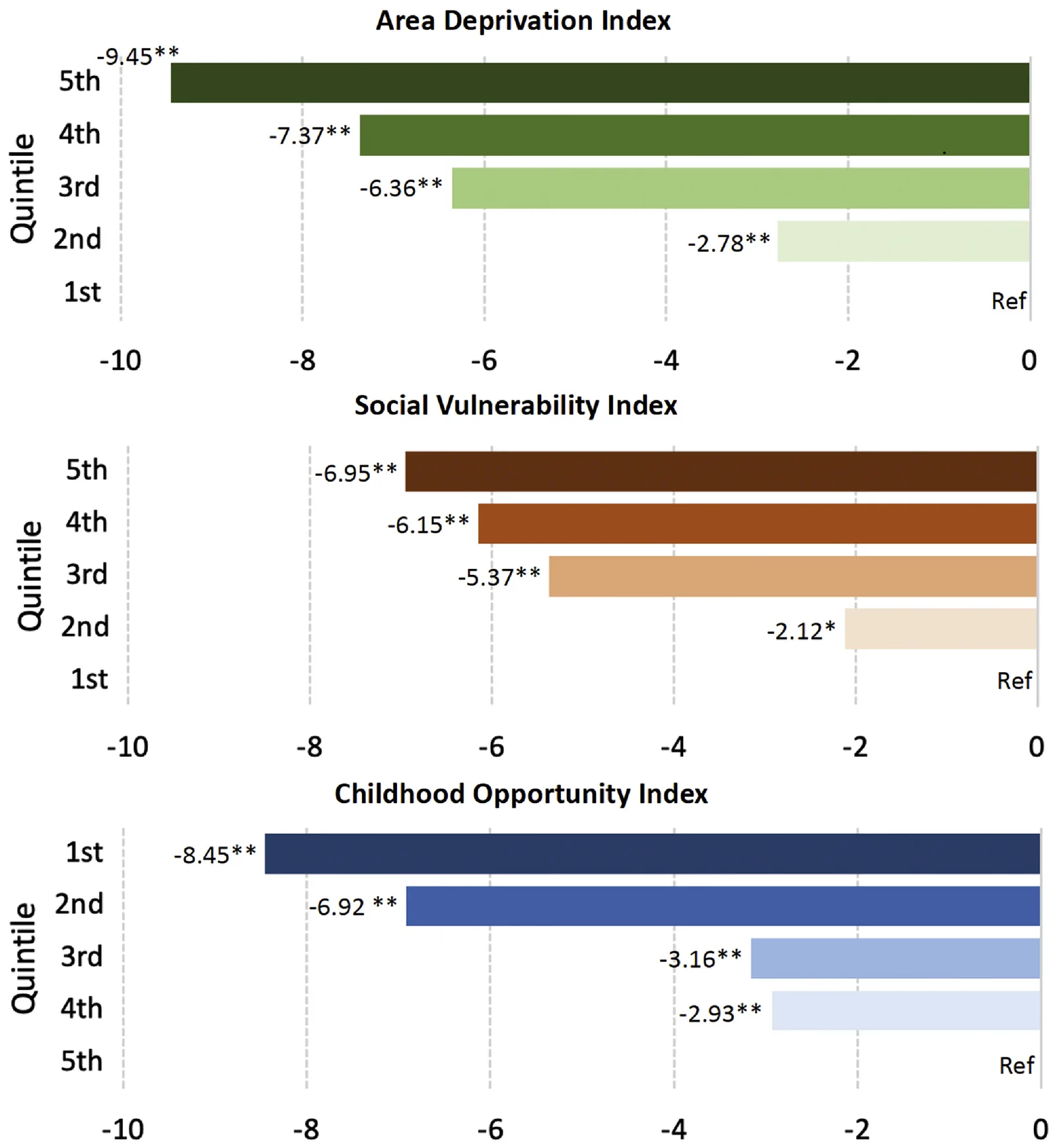
Higher baseline neighborhood disadvantage (high ADI, high SVI, low COI) was associated with higher cardiovascular disease risk (lower Life’s Essential 8 scores) at Years 2 and 3. Statistical significance: *p < 0.01, **p < 0.001. Ref = reference group: “most favorable” neighborhood quality quintile (low ADI, low SVI, high COI).

A significant dose-response relationship was observed for the overall LE8 (N=1,125) and behavioral subcomponent (N=8,160) scores, with these scores progressively decreasing across increasing neighborhood deprivation quintiles for all three indices (ADI, SVI, and COI) (p-trend < 0.001). Although statistically significant, the linear regression models only explained a modest proportion of variance in LE8 scores (Supplementary Table S3). Results from modified Poisson regression models were largely consistent with those from multivariable linear regression analyses (Supplementary Table S4).

When examining individual LE8 components (behavioral components: N=8,160; health components: N=1,125), neighborhood disadvantage was significantly associated with lower diet, nicotine, physical activity, sleep duration, and BMI scores (Supplementary Table S5).

## 4 Discussion

Our study found that higher neighborhood disadvantage across all three indices (ADI, SVI, and COI) showed approximately dose-response relationships with lower LE8 cardiovascular health scores in early adolescents in a large, nationally representative cohort study. Specifically, early adolescents in the most advantaged neighborhoods had LE8 scores 7–9 points higher than those in the most disadvantaged neighborhoods. A 10-point increase in LE8 score has been associated with a 22–40% reduction in CVD risk [27]; therefore, while caution is warranted when extrapolating to adolescents, the 7–9-point lower scores observed among early adolescents in the most disadvantaged neighborhoods may have clinically meaningful implications for future cardiovascular risk. The consistency of findings across ADI, SVI, and COI also suggests an association between neighborhood context and LE8 scores. This consistency potentially reflects shared underlying dimensions of neighborhood disadvantage captured across these indices (e.g., socioeconomic status), although each measure also incorporates distinct conceptual domains.

The results also demonstrated that associations between neighborhood disadvantage and LE8 scores were primarily driven by behavioral factors (e.g., diet, physical activity, nicotine exposure, sleep duration), with strong and consistent associations observed for behavioral factors and largely nonsignificant associations with health factors. Additionally, confidence intervals for health factor (physiological health) estimates were relatively wide, suggesting limited precision and warranting caution in interpretation. Behavioral factors may drive the relationship between overall LE8 cardiovascular health scores and neighborhood disadvantage because, in early adolescence, CVD risk is more immediately shaped by modifiable behaviors such as physical activity and healthy sleep than by cardiometabolic measures like BMI, lipids, glucose, and blood pressure, which accumulate with aging and manifest more strongly over time. This suggests that neighborhood environments shape cardiovascular health in early adolescence primarily by enabling or limiting healthy behaviors through access to recreational spaces, healthy food options, and environments that facilitate quality sleep.

These findings align with a growing body of literature connecting neighborhood disadvantage to cardiovascular disease risk [28]. Previous studies have found associations between neighborhood disadvantage and CVD in adults [9,[29], [30], [31], [32], [33]], and area deprivation and cardiometabolic health among children and young adults [34,35]. However, no study to our knowledge has examined cardiovascular health using the LE8 framework in early adolescence in conjunction with three measures of neighborhood disadvantage.

Our study has several key strengths, including the use of a large, geographically diverse national cohort, the incorporation of multiple, validated measures of neighborhood-level deprivation, and a comprehensive assessment of cardiovascular health using the AHA’s LE8 framework. Limitations of our study include reliance on self- and caretaker-reported behavioral subcomponent data and potential residual confounding by unmeasured family or individual characteristics. Another limitation is that neighborhood disadvantage was measured at a single time point, limiting our ability to assess cumulative or time-varying neighborhood exposures. Given the examination of multiple exposures and outcomes, there is a potential for inflation of Type I error, which should be considered when interpreting the findings. Additionally, some cardiometabolic measures were obtained from either the Year 2 or Year 3 follow-up due to COVID-19-related disruptions in data collection. Consequently, outcomes were not measured at a uniform time point across participants, introducing the potential for timing-related variability and selection bias. However, incorporating available Year 3 data improved completeness of cardiometabolic measures and reduced the loss of participants that would have occurred if analyses were restricted to Year 2 data alone. Participants excluded from the analytic sample for LE8 scores were more likely to be younger and Black compared with those included, potentially resulting in underrepresentation of these groups and limiting the generalizability of the findings. Furthermore, behavioral and health factor estimates were not directly comparable due to differing analytic samples.

These findings have notable clinical and policy implications. Clinicians caring for early adolescents should consider integrating neighborhood disadvantage into cardiovascular risk assessment. Neighborhood disadvantage indices, such as ADI, could be integrated into electronic health records to identify patients who may benefit from social needs screening, connections to social workers, and additional resources. Our studies also reinforce recent calls for a Health in All Policies approach to urban and community planning [36]. This policy framework prioritizes neighborhood features such as walkable communities, access to healthy food, and green spaces, which have been shown to improve health outcomes by addressing barriers to healthful behaviors. This framework aligns with our finding that neighborhood disadvantage shapes early adolescent cardiovascular health through behavioral pathways. When young people have access to sidewalks, parks, and healthy foods within their schools and neighborhoods, they are more likely to adopt behaviors that reduce their risk of CVD.

Central illustration

**Figure:**
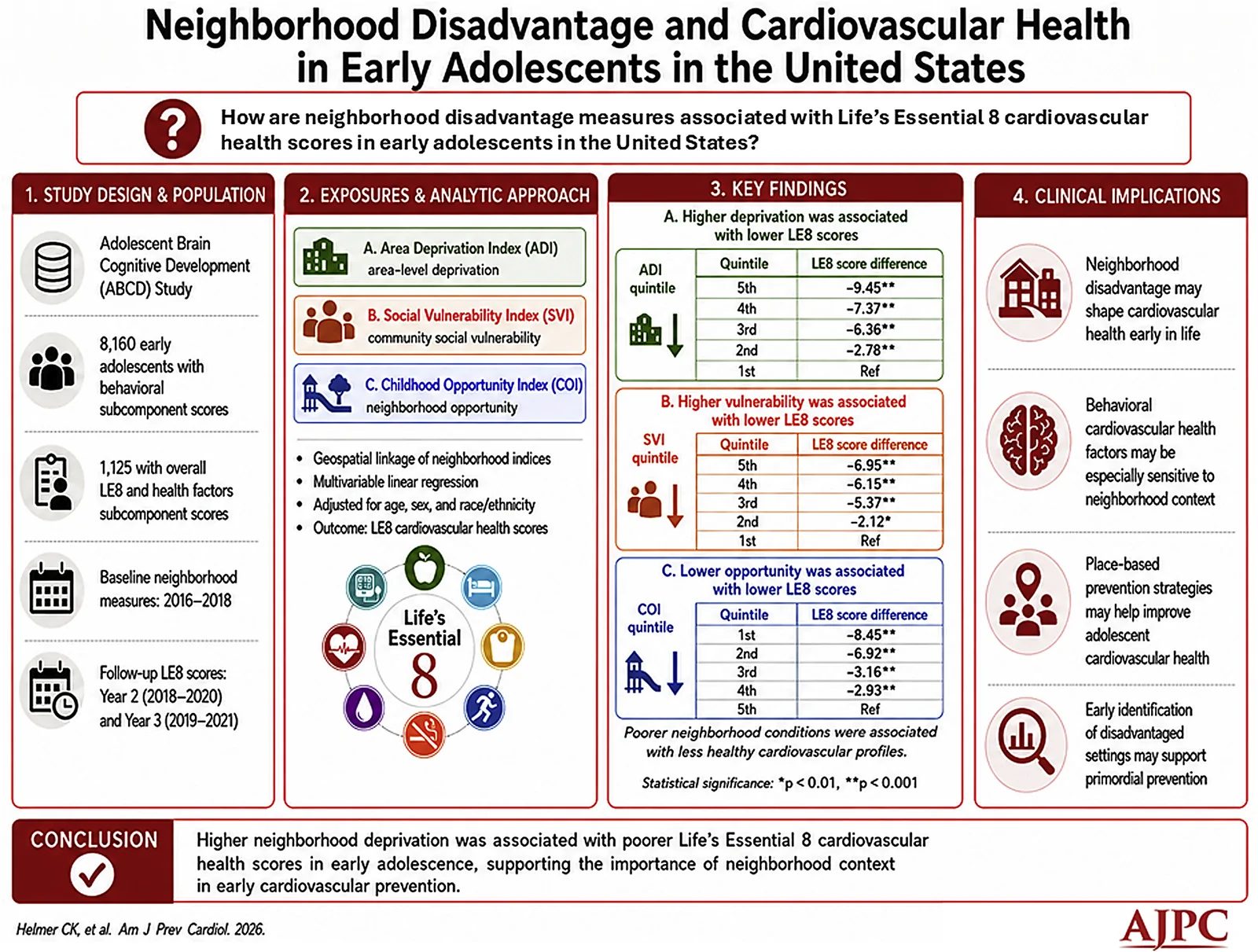

## 5 Conclusion

Adolescence is a critical developmental period when exposure to neighborhood disadvantage may initiate pathophysiological processes that culminate in adult CVD. This study demonstrates that higher neighborhood-level deprivation, as measured by ADI, SVI, and COI, is independently associated with CVD risk factors, particularly behavioral factors and BMI, in early adolescence. Understanding the connection between neighborhood resource access and cardiovascular risk across the life course could enable physicians and health systems to target high-risk neighborhoods for intensive primary prevention strategies that address upstream determinants of CVD.

## Declaration of generative AI and AI-assisted technologies in the manuscript preparation process

During the preparation of this work, the authors used ChatGPT to assist in the creation of the central illustration. The generated output was reviewed and, where necessary, modified by the authors. The authors take full responsibility for the content of the published article.

## Acknowledgements

### Funding

J.M.N. was funded by the National Institutes of Health (K08HL1549350) and the Doris Duke Charitable Foundation (2022056). The funders had no role in the design and conduct of the study; collection, management, analysis, and interpretation of the data; preparation, review, or approval of the manuscript; and decision to submit the manuscript for publication.

The ABCD Study was supported by the National Institutes of Health and additional federal partners under award numbers U01DA041022, U01DA041025, U01DA041028, U01DA041048, U01DA041089, U01DA041093, U01DA041106, U01DA041117, U01DA041120, U01DA041134, U01DA041148, U01DA041156, U01DA041174, U24DA041123, and U24DA041147. A full list of supporters is available at https://abcdstudy.org/federal-partners/. A listing of participating sites and a complete listing of the study investigators can be found at https://abcdstudy.org/principal-investigators.html. ABCD consortium investigators designed and implemented the study and/or provided data but did not necessarily participate in the analysis or writing of this report.

### Ethics approval

The University of California, San Diego provided centralized institutional review board (IRB) approval and each participating site received local IRB approval.

### Consent, data, materials and/or code availability

Written informed consent and assent were obtained from the parent/guardian and adolescent, respectively, to participate in the ABCD Study. Data used in the preparation of this article were obtained from the ABCD Study (https://abcdstudy.org), held in the held in the NIH Brain Development Cohorts (NBDC) Portal.

## CRediT authorship contribution statement

**Christiane K. Helmer:** Writing – review & editing, Writing – original draft, Formal analysis. **Olivia Veira:** Writing – review & editing, Writing – original draft, Formal analysis. **Erin E. Dooley:** Writing – review & editing. **Hoda S. Abdel Magid:** Writing – review & editing. **Alexander Testa:** Writing – review & editing. **Kelley Pettee Gabriel:** Writing – review & editing. **Fiona C. Baker:** Writing – review & editing, Methodology, Data curation. **Holly C. Gooding:** Writing – review & editing. **Jason M. Nagata:** Writing – review & editing, Writing – original draft, Supervision, Formal analysis, Data curation, Conceptualization.

## Declaration of competing interest

The authors declare that they have no known competing financial interests or personal relationships that could have appeared to influence the work reported in this paper.
