# Supplementary material for: Neighborhood disadvantage and cardiovascular health in early adolescents in the United States
Source: Am J Prev Cardiol. 2026 Jul 17;30:101749. doi: 10.1016/j.ajpc.2026.101749 (PMC13633921; doi:10.1016/j.ajpc.2026.101749)
Supplement: Supplementary file 1 [file mmc1.docx]

| Supplementary Table S1. Comparison of the sociodemographic characteristics of the Adolescent Brain Cognitive Development (ABCD) Study participants included vs. excluded in the behavioral factors analysis | | | |
| --- | --- | --- | --- |
| Sociodemographic characteristics | Included (n=8,160) | Excluded (n=3,802) | p |
| Age (years) | 9.9 (0.6) | 9.9 (0.6) | <0.001 |
| Sex |  |  | 0.059 |
| Female | 47.3% | 49.2% |  |
| Male | 52.7% | 50.8% |  |
| Race and ethnicity |  |  | <0.001 |
| Asian | 6.2% | 5.5% |  |
| Black | 15.9% | 29.9% |  |
| Latino/Hispanic | 15.0% | 21.9% |  |
| Native American | 3.5% | 3.4% |  |
| Other | 0.9% | 1.0% |  |
| White | 58.4% | 38.3% |  |
|  |  |  |  |
|  |  |  |  |
|  |  |  |  |
|  |  |  |  |
|  |  |  |  |
|  |  |  |  |

| Supplementary Table S2. Comparison of the sociodemographic characteristics of the Adolescent Brain Cognitive Development (ABCD) Study participants included vs. excluded in the overall Life's Essential 8 (LE8) and health factors analysis | | | |
| --- | --- | --- | --- |
| Sociodemographic characteristics | Included (n=1,125) | Excluded (n=10,837) | p |
| Age (years) | 10.0 (0.6) | 9.9 (0.6) | 0.005 |
| Sex |  |  | 0.273 |
| Female | 46.3% | 48.0% |  |
| Male | 53.7% | 52.0% |  |
| Race and ethnicity |  |  | <0.001 |
| Asian | 4.3% | 6.2% |  |
| Black | 12.0% | 21.1% |  |
| Latino/Hispanic | 13.4% | 17.6% |  |
| Native American | 4.1% | 3.4% |  |
| Other | 0.9% | 0.9% |  |
| White | 65.3% | 50.8% |  |

| Supplementary Table S3. Linear regression model fit (R^2^) for associations of baseline indices of children's neighborhood quality and Life's Essential 8 (LE8) cardiovascular health characteristics at Year 2 and 3 in the Adolescent Brain Cognitive Development (ABCD) Study | | | |
| --- | --- | --- | --- |
| Children's neighborhood quality | Overall LE8 summary score | Behavioral factors subcomponent score | Health factors subcomponent score |
|  | N=1,125 | N=8,160 | N=1,125 |
|  | R^2^ | R^2^ | R^2^ |
| Area Deprivation Index | 0.1590 | 0.1388 | 0.0531 |
| Social Vulnerability Index | 0.1388 | 0.1076 | 0.0626 |
| Child Opportunity Index | 0.1415 | 0.1226 | 0.0543 |
| Linear regression models were adjusted for age, sex, and race and ethnicity. | | | |

| Supplementary Table S4. Associations of baseline indices of children's neighborhood quality and Life's Essential 8 (LE8) cardiovascular health characteristics at Year 2 and 3 in the Adolescent Brain Cognitive Development (ABCD) Study | | | | | | |
| --- | --- | --- | --- | --- | --- | --- |
| Children's neighborhood quality | Overall LE8 summary score | | Behavioral factors subcomponent score | | Health factors subcomponent score | |
|  | N=1,125 | | N=8,160 | | N=1,125 | |
|  | Adjusted RR (95% CI) | p | Adjusted RR (95% CI) | p | Adjusted RR (95% CI) | p |
| Area Deprivation Index quintile |  |  |  |  |  |  |
| 1st (lowest) | reference |  | reference |  | reference |  |
| 2nd | **1.37 (1.13, 1.67)** | **0.002** | **1.11 (1.07, 1.16)** | **<0.001** | 1.46 (0.99, 2.15) | 0.053 |
| 3rd | **1.77 (1.46, 2.15)** | **<0.001** | **1.27 (1.22, 1.32)** | **<0.001** | **1.83 (1.23, 2.72)** | **0.003** |
| 4th | **1.79 (1.44, 2.22)** | **<0.001** | **1.36 (1.30, 1.42)** | **<0.001** | **1.68 (1.05, 2.69)** | **0.029** |
| 5th (highest) | **1.96 (1.59, 2.42)** | **<0.001** | **1.39 (1.33, 1.45)** | **<0.001** | **1.79 (1.11, 2.88)** | **0.017** |
| Social Vulnerability Index quintile |  |  |  |  |  |  |
| 1st (lowest) | reference |  | reference |  | reference |  |
| 2nd | 1.14 (0.95, 1.36) | 0.165 | 1.04 (1.00, 1.09) | 0.049 | 1.33 (0.91, 1.95) | 0.140 |
| 3rd | **1.52 (1.28, 1.81)** | **<0.001** | **1.18 (1.13, 1.23)** | **<0.001** | **2.14 (1.50, 3.06)** | **<0.001** |
| 4th | **1.70 (1.43, 2.03)** | **<0.001** | **1.22 (1.17, 1.27)** | **<0.001** | **1.81 (1.21, 2.72)** | **0.004** |
| 5th (highest) | **1.70 (1.42, 2.03)** | **<0.001** | **1.26 (1.21, 1.31)** | **<0.001** | **2.10 (1.40, 3.15)** | **<0.001** |
| Child Opportunity Index quintile |  |  |  |  |  |  |
| 5th (highest) | reference |  | reference |  | reference |  |
| 4th | **1.32 (1.12, 1.55)** | **0.001** | **1.16 (1.12, 1.21)** | **<0.001** | 1.19 (0.85, 1.67) | 0.306 |
| 3rd | **1.35 (1.12, 1.63)** | **0.002** | **1.24 (1.19, 1.29)** | **<0.001** | 1.31 (0.89, 1.92) | 0.172 |
| 2nd | **1.72 (1.45, 2.05)** | **<0.001** | **1.30 (1.24, 1.35)** | **<0.001** | **1.74 (1.21, 2.49)** | **0.003** |
| 1st (lowest) | **1.82 (1.52, 2.17)** | **<0.001** | **1.35 (1.30, 1.41)** | **<0.001** | **1.60 (1.05, 2.42)** | **0.028** |
| **Bold** indicates statistical significance after the Benjamini-Hochberg procedure. RR = risk ratio. Modified Poisson regression models were adjusted for age, sex, and race and ethnicity. Reference group: “most favorable” neighborhood quality quintile (low ADI, low SVI, high COI) and high LE8 score (80–100). The four behavioral factors include diet, physical activity, nicotine exposure, and sleep; the four health factors include body mass index, blood lipids, blood glucose, and blood pressure. | | | | | | |

| Supplementary Table S5. Associations of baseline indices of children's neighborhood quality and individual Life's Essential 8 (LE8) cardiovascular health characteristics at Year 2 and 3 in the Adolescent Brain Cognitive Development (ABCD) Study | | | | | | | | |
| --- | --- | --- | --- | --- | --- | --- | --- | --- |
| Children's neighborhood quality | Behavioral factors (N=8,160) | | | | | | | |
|  | Diet score | | Nicotine exposure score | | Physical activity score | | Sleep duration score | |
|  | Adjusted B (95% CI) | p | Adjusted B (95% CI) | p | Adjusted B (95% CI) | p | Adjusted B (95% CI) | p |
| Area Deprivation Index quintile |  |  |  |  |  |  |  |  |
| 1st (lowest) | reference |  | reference |  | reference |  | reference |  |
| 2nd | **-3.04 (-4.72, -1.36)** | **<0.001** | -0.11 (-0.53, 0.31) | 0.604 | **-5.93 (-8.18, -3.68)** | **<0.001** | -0.97 (-2.21, 0.27) | 0.126 |
| 3rd | **-10.03 (-12.02, -8.03)** | **<0.001** | **-1.00 (-1.49, -0.50)** | **<0.001** | **-13.94 (-16.62, -11.27)** | **<0.001** | **-3.61 (-5.09, -2.14)** | **<0.001** |
| 4th | **-16.08 (-18.53, -13.64)** | **<0.001** | **-2.41 (-3.01, -1.80)** | **<0.001** | **-18.52 (-21.79, -15.24)** | **<0.001** | **-7.63 (-9.43, -5.82)** | **<0.001** |
| 5th (highest) | **-17.73 (-20.30, -15.17)** | **<0.001** | **-2.55 (-3.19, -1.92)** | **<0.001** | **-23.65 (-27.09, -20.21)** | **<0.001** | **-8.05 (-9.95, -6.16)** | **<0.001** |
| Social Vulnerability Index quintile |  |  |  |  |  |  |  |  |
| 1st (lowest) | reference |  | reference |  | reference |  | reference |  |
| 2nd | -1.44 (-3.22, 0.34) | 0.113 | -0.47 (-0.91, -0.04) | 0.034 | -1.58 (-3.94, 0.78) | 0.190 | -1.21 (-2.51, 0.10) | 0.069 |
| 3rd | **-6.22 (-8.21, -4.23)** | **<0.001** | **-0.61 (-1.10, -0.12)** | **0.015** | **-8.71 (-11.36, -6.06)** | **<0.001** | **-3.35 (-4.81, -1.89)** | **<0.001** |
| 4th | **-8.11 (-10.30, -5.91)** | **<0.001** | **-1.14 (-1.68, -0.60)** | **<0.001** | **-14.31 (-17.22, -11.39)** | **<0.001** | **-5.38 (-6.99, -3.77)** | **<0.001** |
| 5th (highest) | **-9.16 (-11.52, -6.81)** | **<0.001** | **-1.44 (-2.02, -0.87)** | **<0.001** | **-19.10 (-22.23, -15.97)** | **<0.001** | **-5.75 (-7.48, -4.03)** | **<0.001** |
| Child Opportunity Index quintile |  |  |  |  |  |  |  |  |
| 5th (highest) | reference |  | reference |  | reference |  | reference |  |
| 4th | **-4.78 (-6.47, -3.09)** | **<0.001** | -0.21 (-2.41, -1.24) | 0.334 | **-7.46 (-9.71, -5.21)** | **<0.001** | **-2.31 (-3.55, -1.07)** | **<0.001** |
| 3rd | **-6.62 (-8.68, -4.56)** | **<0.001** | **-0.90 (-1.41, 0.39)** | **<0.001** | **-11.08 (-13.83, -8.34)** | **<0.001** | **-3.71 (-5.22, -2.20)** | **<0.001** |
| 2nd | **-10.14 (-12.49, -7.79)** | **<0.001** | **-1.72 (-2.30, -1.15)** | **<0.001** | **-16.71 (-19.84, -13.58)** | **<0.001** | **-8.21 (-9.93, -6.48)** | **<0.001** |
| 1st (lowest) | **-13.84 (-16.23, -11.45)** | **<0.001** | **-1.83 (-2.41, -1.24)** | **<0.001** | **-24.13 (-27.31, -20.96)** | **<0.001** | **-6.86 (-8.61, -5.11)** | **<0.001** |
| Children's neighborhood quality | Health factors (N=1,125) | | | | | | | |
|  | Body mass index score | | Blood lipids score | | Blood glucose score | | Blood pressure score | |
|  | Adjusted B (95% CI) | p | Adjusted B (95% CI) | p | Adjusted B (95% CI) | p | Adjusted B (95% CI) | p |
| Area Deprivation Index quintile |  |  |  |  |  |  |  |  |
| 1st (lowest) | reference |  | reference |  | reference |  | reference |  |
| 2nd | -3.29 (-7.68, 1.09) | 0.141 | -0.11 (-4.41, 4.18) | 0.959 | -0.11 (-1.24, 1.03) | 0.856 | -1.46 (-3.24, 0.33) | 0.109 |
| 3rd | **-9.09 (-14.07, -4.11)** | **<0.001** | -0.28 (-5.16, 4.59) | 0.910 | -0.68 (-1.97, 0.61) | 0.302 | -0.76 (-2.79, 1.27) | 0.463 |
| 4th | **-8.48 (-14.84, -2.12)** | **0.009** | 1.37 (-4.86, 7.59) | 0.667 | -0.17 (-1.82, 1.48) | 0.836 | 0.93 (-1.66, 3.52) | 0.479 |
| 5th (highest) | **-15.05 (-22.05, -8.04)** | **<0.001** | 4.90 (-1.96, 11.75) | 0.161 | -1.43 (-3.25, 0.39) | 0.123 | 1.57 (-1.28, 4.42) | 0.280 |
| Social Vulnerability Index quintile |  |  |  |  |  |  |  |  |
| 1st (lowest) | reference |  | reference |  | reference |  | reference |  |
| 2nd | -2.13 (-6.36, 2.10) | 0.323 | -3.82 (-7.99, 0.35) | 0.073 | -0.40 (-1.51, 0.71) | 0.477 | 0.02 (-1.72, 1.76) | 0.983 |
| 3rd | **-11.74 (-16.63, -6.85)** | **<0.001** | -1.98 (-6.81, 2.84) | 0.420 | -0.72 (-2.00, 0.56) | 0.271 | -0.81 (-2.82, 1.21) | 0.433 |
| 4th | **-10.64 (-16.31, -4.98)** | **<0.001** | 2.06 (-3.53, 7.65) | 0.469 | -0.07 (-1.56, 1.41) | 0.922 | 0.19 (-2.14, 2.53) | 0.871 |
| 5th (highest) | **-17.41 (-23.48 -11.34)** | **<0.001** | 1.54 (-4.45, 7.53) | 0.615 | -1.10 (-2. 70, 0.49) | 0.174 | 0.06 (-2.44, 2.56) | 0.963 |
| Child Opportunity Index quintile |  |  |  |  |  |  |  |  |
| 5th (highest) | reference |  | reference |  | reference |  | reference |  |
| 4th | -1.94 (-6.08, 2.19) | 0.356 | -4.41 (-8.46, -0.35) | 0.033 | -0.50 (-1.57, 0.58) | 0.367 | 1.32 (-0.37, 3.01) | 0.126 |
| 3rd | -5.06 (-10.12, -0.01) | 0.050 | 0.52 (-4.43, 5.48) | 0.836 | 0.27 (-1.05, 1.58) | 0.689 | -0.85 (-2.91, 1.22) | 0.422 |
| 2nd | **-11.95 (-17.67, -6.24)** | **<0.001** | 1.94 (-3.66, 7.54) | 0.497 | -1.27 (-2.75, 0.22) | 0.094 | 0.63 (-1.70, 2.97) | 0.595 |
| 1st (lowest) | **-16.73 (-23.40, -10.06)** | **<0.001** | 1.69 (-4.85, 8.22) | 0.613 | -1.33 (-3.07, 0.40) | 0.133 | 2.22 (-0.50, 4.95) | 0.110 |
| **Bold** indicates statistical significance after the Benjamini-Hochberg procedure. Linear regression models were adjusted for age, sex, and race and ethnicity. | | | | | | | | |
